# MUC18 Regulates Lung Rhinovirus Infection and Inflammation

**DOI:** 10.1371/journal.pone.0163927

**Published:** 2016-10-04

**Authors:** Reena Berman, Di Jiang, Qun Wu, Connor R. Stevenson, Niccolette R. Schaefer, Hong Wei Chu

**Affiliations:** Department of Medicine, National Jewish Health, 1400 Jackson Street, Denver, Colorado 80206, United States of America; University of Alabama at Birmingham, UNITED STATES

## Abstract

**Background:**

MUC18 is upregulated in the lungs of asthma and COPD patients. It has been shown to have pro-inflammatory functions in cultured human airway epithelial cells during viral infections and in mice during lung bacterial infections. However, the in vivo role of MUC18 in the context of viral infections remains poorly understood. The goal of this study is to define the in vivo function of MUC18 during respiratory rhinovirus infection.

**Methods:**

Muc18 wild-type (WT) and knockout (KO) mice were infected with human rhinovirus 1B (HRV-1B) and sacrificed after 1 day to determine the inflammatory and antiviral responses. To examine the direct effects of Muc18 on viral infection, tracheal epithelial cells isolated from WT and KO mice were grown under air-liquid interface and infected with HRV-1B. Finally, siRNA mediated knockdown of MUC18 was performed in human airway epithelial cells (AECs) to define the impact of MUC18 on human airway response to HRV-1B.

**Results:**

Both viral load and neutrophilic inflammation were significantly decreased in Muc18 KO mice compared to WT mice. In the in vitro setting, viral load was significantly lower and antiviral gene expression was higher in airway epithelial cells of Muc18 KO mice than the WT mice. Furthermore, in MUC18 knockdown human AECs, viral load was decreased and antiviral gene expression was increased compared to controls.

**Conclusions:**

Our study is the first to demonstrate MUC18’s pro-inflammatory and pro-viral function in an in vivo mouse model of rhinovirus infection.

## Introduction

MUC18, also referred to as CD146 or melanoma cell adhesion molecule (MCAM), is a 113 kD transmembrane glycoprotein of the immunoglobulin superfamily [1, 2]. MUC18 is comprised of an extracellular domain, a single transmembrane domain, and a short (63 amino acids) cytoplasmic tail [3]. It is upregulated in the airways of asthmatics and patients with chronic obstructive pulmonary disease (COPD), compared to healthy controls [4].

MUC18 has been previously demonstrated to have pro-inflammatory functions in human airway epithelial cells [3] as well as mouse lungs during bacterial infections [5]. In an over-expression model of MUC18 in human airway epithelial cells with human rhinovirus (HRV) infection, MUC18 suppressed the expression of antiviral genes and promoted production of the pro-inflammatory cytokine IL-8 [3]. However, the in vivo role of MUC18 in viral infections, particularly in the context of HRV, has not yet been determined.

HRV infection is the major contributor to exacerbations of various lung diseases including asthma and COPD. A common characteristic of exacerbations of lung diseases is excessive inflammation, demonstrated by increases in neutrophils and IL-8, a chemoattractant of neutrophils. In our previous publication [3], we showed that MUC18 promotes IL-8 production in human airway epithelial cells. A study by Gern et al showed that IL-8 was rapidly induced after viral inoculation and contributed to neutrophil trafficking in the human upper airways [6]. In addition to IL-8, the production of other inflammatory markers is considered to be an indicator of infection. Interferon-γ-Inducible Protein 10 (IP-10 or CXCL10) is produced by human airway epithelial cells in response to rhinovirus infections and elevated in BAL fluid of patients with respiratory virus compared to healthy controls [7, 8]. However, the role of MUC18 in neutrophil recruitment during lung (in vivo) HRV infection has yet to be investigated.

Using a knockout (KO) mouse model of Muc18, we sought to determine a role of Muc18 in viral infections in vivo. We hypothesized that MUC18/Muc18 promotes lung viral infections and inflammation. We expect that during viral infection, Muc18 KO mice will have greater expression of antiviral genes and subsequently less pro-inflammatory responses such as neutrophil recruitment. Furthermore, we utilized mouse and human airway epithelial cell culture system to determine the underlying mechanisms for MUC18’s in vivo functions during rhinovirus infection.

## Methods

### Mice

The Institutional Animal Care and Use Committee (IACUC) of National Jewish Health approved our use of mice under protocol AS2792-04-17. Muc18^+/-^ mice on 129SvEvBr background were obtained from Taconic Farms (Hudson, NY: distributed through Lexicon Pharmaceuticals, The Woodlands, TX). Muc18^+/+^ (wild-type, WT) and Muc18^-/-^ (knockout, KO) were generated by breeding Muc18^+/-^ mice in our biological resource center under pathogen-free housing conditions [5]. Animals were monitored daily for their ability to move, as well as changes in behavior, activity, or posture, and showed no signs of wounds, significant (>20%) body weight loss, or other indicators of disease.

### Human Rhinovirus Preparation and Infection in Mice

HRV-1B (American Type Culture Collection, Manassas, VA) was propagated in H1-HeLa cells (CRL-1958, ATCC), purified, and titrated as described previously [9]. MUC18 WT and KO (8–12 weeks of age) mice were anesthetized by intraperitoneal injection of ketamine (70mg/kg) and xylazine (10 mg/kg), and were intranasally inoculated with 50μL of phosphate buffered saline (PBS) as a control or HRV-1B at 1x10^7^ plaque-forming units (pfu) per mouse. Mice were sacrificed 1 day after HRV-1B infection or PBS treatment. The 1 day time-point in our in vivo model was selected because previously published research [10] and our own time course experiments suggest that both lung viral load and lung inflammation peak on 1 day post infection, but significantly decreased at both days 3 and 7 post infection.

### Mouse Bronchoalveolar Lavage (BAL) and Lung Tissue Processing

Mice were euthanized by intraperitoneal injection of pentobarbital sodium (FATAL Plus, VORTECH PHARMACEUTICALS, Dearborn, MI) in sodium chloride. Lungs were lavaged with 1 mL of saline solution. Cell-free BAL fluid was stored at -80°C for cytokine analysis. BAL cell cytospin slides were stained with a Diff-Quick stain kit (IMEB, San Marcos, CA) for cell differential counts. Leukocyte differentials were determined as percentage of 500 counted leukocytes. Left lungs and cell-free BAL were used for RNA extraction and quantitative real-time PCR.

### Mouse Tracheal Epithelial Cell Isolation and Air-Liquid Interface Culture

Tracheas from PBS treated (Control) groups were pooled by group and digested overnight in 0.1% protease (Sigma-Aldrich, St. Louis, MO) in 1x Pen/Strep/Amphoteracin solution (MP Biomedicals, Solon, OH)/DMEM stock solution (HyClone, Logan, UT). Epithelial cells were washed off the trachea in a 2% Fetal Bovine Solution/DMEM medium and incubated for 4 hours to allow debris to adhere to the dish. Cells were then seeded onto transwell inserts and cultured for one week before being shifted to air liquid interface (ALI) as previously described [11]. On the 10^th^ day of ALI, cells were infected with HRV-1B at 5x10^4^ 50% Tissue Culture Infective Dose (TCID_50_) as previously described and harvested 24 hours later.

### Human Tracheobronchial Epithelial Cell Culture

Human cell samples for this study were obtained from the tracheas and bronchi of de-identified donors whose lungs were not suitable for transplantation and no lung diseases were identified. These samples were obtained through the Mucosal Immunity Research Program at National Jewish Health, in collaboration with the Donor Alliance Organ and Tissue Donation network. Donors previously provided written consent to donate to the Donor Alliance prior to death, or consent was obtained from next of kin immediately following the death. The Institutional Review Board at National Jewish Health approved collection and use of these cells [12]. Airway epithelial cells at passage 1 were cultured in collagen-coated 60 mm tissue culture dishes containing bronchial epithelial cell growth medium (BEGM) with supplements (Lonza, Walkersville, MD) at 37°C, 5% CO_2_, until 90% confluence. Epithelial cells at passage 2 were seeded into 12-well culture plates at 1.7x10^5^ cells/well.

### siRNA Mediated MUC18 Knockdown (KD) and HRV Infection in Human Tracheobronchial Epithelial Cells

Cells were transfected with either MUC18 siRNA or scrambled control siRNA with no homology to any known gene sequence by incubating the cells with the mixture of a transfection reagent and MUC18 or control siRNA (Santa-Cruz Biotechnology, Dallas, TX) for 6 hours followed by removing the transfection medium and refreshing the cells with BEGM. After 24 hours of siRNA transfection, cells were infected with HRV-1B at 5x10^4^ TCID_50_. Cells were harvested for quantitative RT-PCR at indicated time points (15 minutes, 30 minutes, 2 hours, and 24 hours) after HRV-1B infection. The efficiency of MUC18 knockdown was confirmed via Western blot and densitometry.

### Endosome Staining with LysoSensor Fluorescent Green Dye in Human Tracheobronachial Epithelial Cells with MUC18 Knockdown

The purpose of this assay was to detect viral entry into endosomes under various conditions such as MUC18 knockdown. Thirty minutes prior to the harvest time point, infection medium was replaced with virus plus LysoSensor DND-189 Fluorescent Dye at 5 μM in PBS (ThermoFisher, Waltham, MA) [13, 14]. Cells previously seeded on cover slips in 12 well plates were removed at the end of the 2 hours of viral infection. Cover slips were incubated with DAP1 for 1 minute and then mounted with a fluorescent mounting solution onto microscope slides. Photos were taken using a camera mounted to a fluorescent microscope and the images were overlayed to indicate both blue nuclear staining (with DAP1) and green endosome staining (with LysoSensor).

### Western Blot Analysis

Cells were lysed in RIPA buffer with protease inhibitors (Fisher Scientific, Waltham, MA). The same amount of protein lysate (e.g., 30 μg) was electrophoresed on an 8% SDS-PAGE gel, transferred onto a nitrocellulose membrane, blocked with 2.5% nonfat milk, and incubated with antibodies against MUC18 (Abcam, San Francisco, CA) and GAPDH (Santa Cruz Biotech Inc., Santa Cruz, CA) overnight at 4°C. After washing, membranes were incubated with the appropriate HRP-linked secondary antibodies and Pierce ECL Prime Western blotting substrate (Fisher Scientific, Waltham, MA).

### Enzyme-linked immunosorbent assay (ELISA)

KC levels in mouse BAL fluid and cell culture supernatants were determined by using the mouse KC ELISA DuoSet development kit (R&D Systems, Minneapolis, MN).

### Quantitative Real-Time RT-PCR

TaqMan gene expression assays for Mx1 (Human: Hs00895608_m1, Mouse: Mm00487796_m1), IP-10 (Mouse: Mm00487796_m1), IL-8 (Human: Hs00174103_m1), and IRF-3 (Mouse: Mm00487796_m1) were obtained from Applied Biosystems (Life Technologies, Foster City, CA). Quantitative TaqMan gene expression assays for HRV were custom made from Integrated DNA Technologies (IDT, Coralville, IA). The specific primers and probes were: HRV (forward: 5′-CCT CCG GCC CCT GAA T-3′; reverse: 5′-GGT CCC ATC CCG CAA TT-3′, probe: 5′-CTA ACC TTA AAC CTG CAG CCA-3′). The housekeeping genes, GAPDH (for human samples) and 18s (for mouse samples) (Life Technologies, Foster City, CA) were evaluated as internal positive controls. Quantitative real-time PCR was performed on the CFX96^TM^ real-time PCR Detection System (Bio-Rad, Hercules, CA, USA). The comparative cycle of threshold (ΔΔCt) method was used to demonstrate the relative levels of target genes.

### Statistical analysis

Data are presented as medians or means ± SEMs. One-way nonparametric analysis was used for group comparisons by applying the Wilcoxon test (two group comparison) or the Kruskal-Wallis test (multiple group comparison), where appropriate. One-way ANOVA was used for multiple comparisons and a Tukey post hoc test was applied, where appropriate. The Student t test was used where only 2 groups were compared. A p value of less than 0.05 was considered significant.

## Results

### Muc18 knockout (KO) mice had higher lung antiviral gene expression and lower viral load

**Fig 1:**
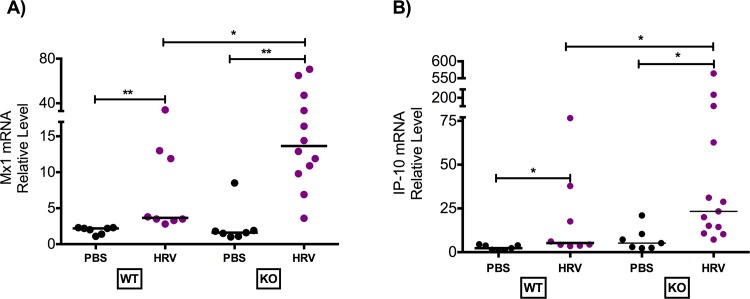
Increased antiviral gene expression in Muc18 knockout (KO) mice infected with human rhinovirus 1B (HRV-1B). Muc18 WT (n = 9) and KO (n = 13) were intranasally infected with HRV-1B or PBS as a control (WT: n = 7; KO: n = 7) and sacrificed 24 hours post treatment. In lung tissue, KO mice had significantly higher expression of the antiviral genes Mx1 **(A)** and IP-10 **(B)** compared to WT mice. Dots represent data from individual of the three independent experiments and the horizontal bars indicate median. A single star (*) indicates p<0.05 and two stars (**) indicates p<0.001.

**Fig 2:**
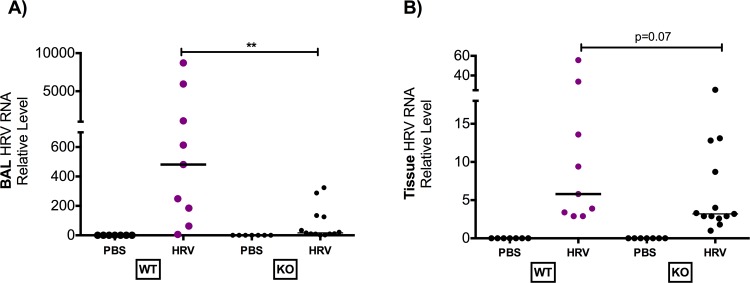
Viral load is significantly reduced in BAL fluid and lung tissue of Muc18 KO mice. Muc18 WT (n = 9) and KO (n = 13) were intranasally infected with HRV-1B or PBS as a control (WT: n = 7; KO: n = 7) and sacrificed 24 hours post treatment. KO mice had significantly lower viral load in BAL fluid than WT mice **(A).** This trend continued in lung tissue **(B)**. Dots represent data from individual of the three independent experiments and the horizontal bars indicate median. A single star (*) indicates p<0.05 and two stars (**) indicates p<0.001.

Viral infection increased lung Mx1 and IP-10 expression in both strains of mice. Muc18 KO mice had significantly higher levels of expression of virus induced Mx1 and interferon-γ-inducible IP-10 than WT mice (Fig 1A and 1B). There was no difference in Mx1 or IP-10 expression level between PBS treated groups. Mx1 is mainly induced by type I interferons such as IFN-α and IFN-β, and exerts antiviral activity [15, 16]. Viral load was significantly lower in BAL fluid of Muc18 KO mice compared to WT mice (Fig 2A). A similar trend was seen in lung tissue, however it was not significant (Fig 2B) (p = 0.07).

### Muc18 KO mice had decreased lung neutrophilic inflammation

**Fig 3:**
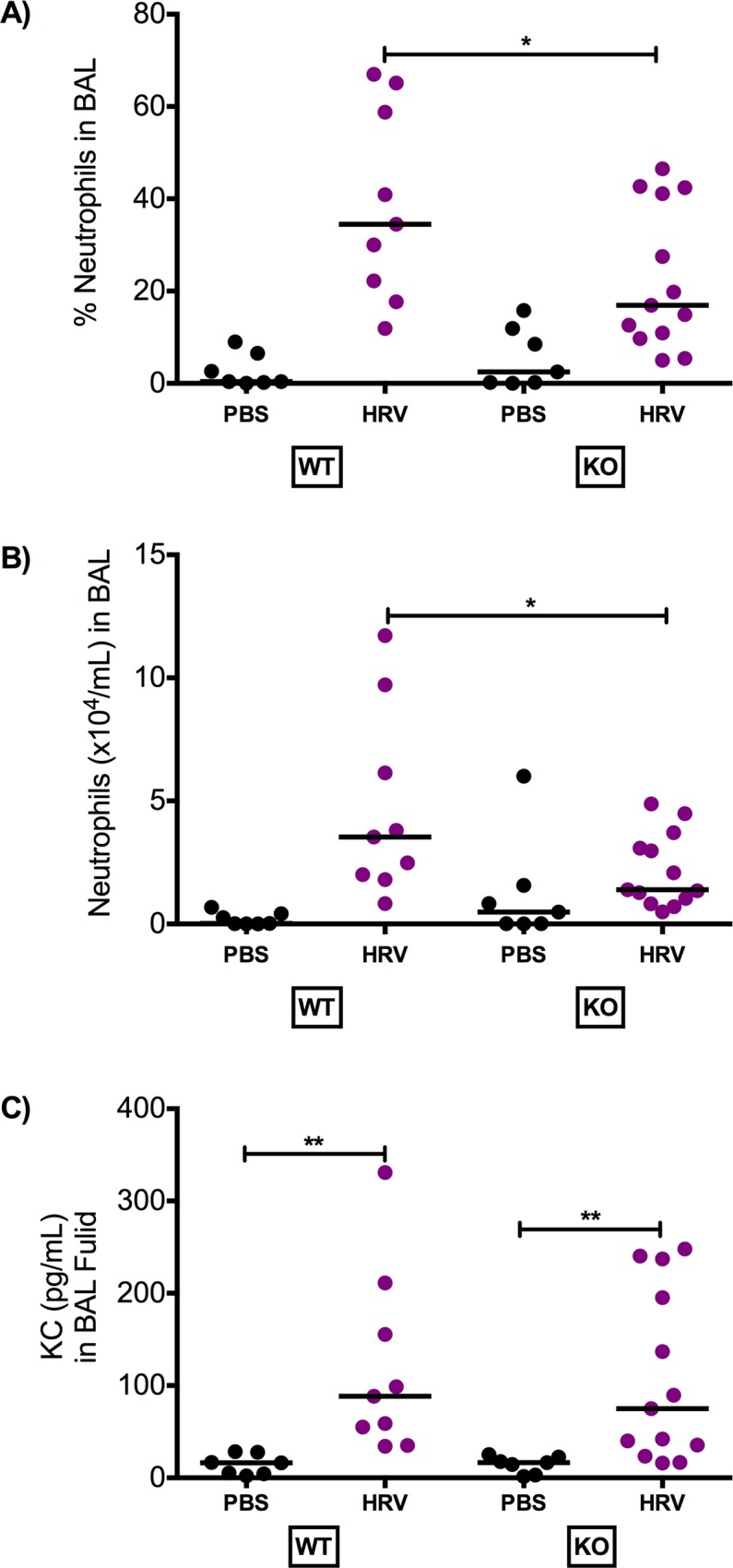
Reduced neutrophils in Muc18 KO mice infected with human rhinovirus 1B (HRV-1B). Muc18 WT (n = 9) and KO (n = 13) were intranasally infected with HRV-1B or PBS as a control (WT: n = 7; KO: n = 7) and sacrificed 24 hours post treatment. Muc18 KO mice had significantly lower levels of neutrophils in bronchoalveolar lavage (BAL) than WT mice. This was seen in both percent neutrophils **(A)** and in absolute number of neutrophils **(B)**. **(C)** HRV infection significantly increased KC levels in WT and KO mice, but there was no significant difference in KC levels between WT and KO mice. Dots represent data from individual of the three independent experiments and the horizontal bars indicate median. The Wilcoxon test was used to compare the difference between groups. A single star (*) indicates p<0.05 and two stars (**) indicates p<0.001.

Compared to WT mice, KO mice had significantly lower percent neutrophils and absolute number of neutrophils in BAL fluid (Fig 3A and 3B). Protein levels of KC, a neutrophil chemoattractant, in BAL fluid increased in response to viral infection in both WT and Muc18 KO mice (Fig 3C), and was consistent with increased neutrophils following one day of viral infection. However, there was no significant difference in KC level between KO and WT mice infected with HRV-1B.

### Cultured tracheal epithelial cells from Muc18 KO mice had lower levels of KC and viral load

**Fig 4:**
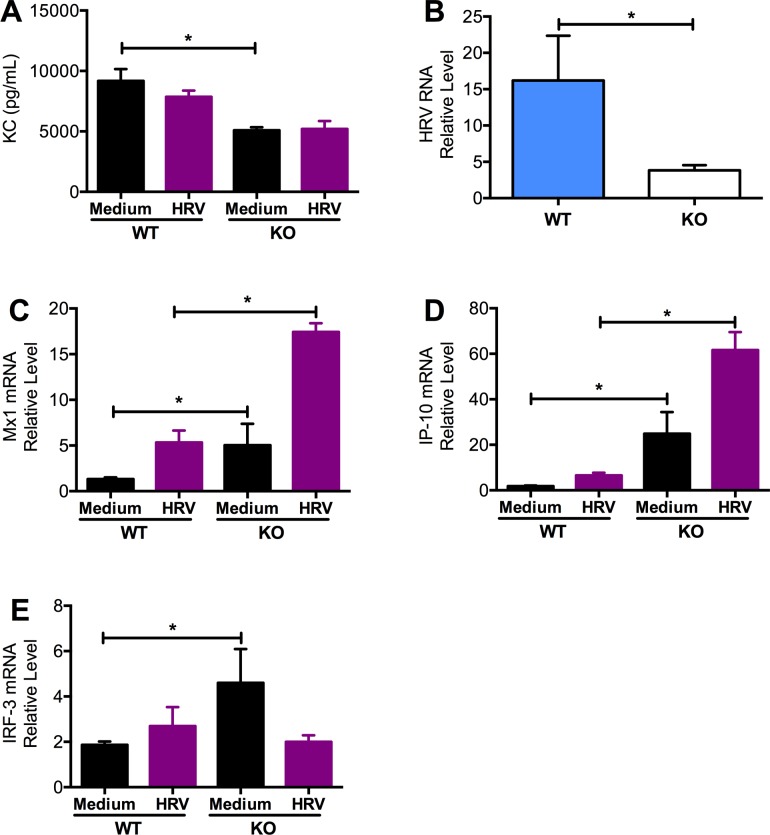
Tracheal epithelial cells of Muc18 KO mice have less KC, less viral load, and greater antiviral gene expression. Muc18 WT (n = 3 replicates) and KO (n = 3 replicates) tracheal epithelial cells were cultured in air-liquid interface and treated with culture medium (–) or infected with HRV-1B for 24 hours. KO cells had significantly less KC **(A)** and viral load **(B)** than WT cells. In KO cells, expression of the antiviral genes Mx1 and IP-10 was significantly greater than WT cells, in both infected and non-infected conditions (p = 0.0495)**(C, D).** IRF-3 gene expression was significantly greater in non-infected KO cells compared to WT (p = 0.0495), but unchanged in infected conditions **(E).** Data are presented as means ± SEMs. A single star (*) indicates p<0.05 and two stars (**) indicates p<0.001.

In an air-liquid interface culture of mouse tracheal epithelial cells, basolateral supernatant from Muc18 KO cells had significantly lower levels of KC compared to WT (Fig 4A). In addition, viral load was significantly lower in KO cells compared to WT cells (Fig 4B). Accompanying this decreased viral load was increased expression of Mx1 (Fig 4C). IP-10 was also upregulated in Muc18 KO cells compared to WT cells (Fig 4D). In ALI culture, IRF-3 was significantly increased in KO mice in the absence of infection, but unchanged when infected with HRV-1B (Fig 4E).

### MUC18 gene knockdown (KD) leads to less viral load in human airway epithelial cells

**Fig 5:**
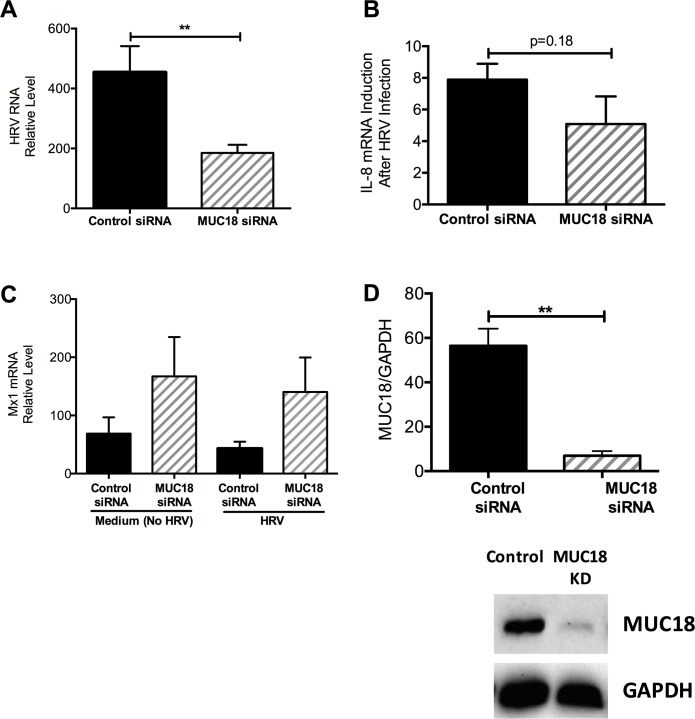
Reduced viral load and IL-8 induction in human airway epithelial cells with MUC18 knockdown (KD). Tracheobronchial epithelial cells from three donors (n = 3 replicates per donor) were transfected with control or MUC18 siRNA and then treated with culture medium (–) or HRV for 2 hours. Cells were harvested 24 hours post infection. Viral load was significantly lower in MUC18 KD cells compared to control **(A).** Expression of the antiviral gene MX1 was similar between KD and control cells **(B).** There was less induction of the pro-inflammatory cytokine, IL-8, in MUC18 KD samples compared to control **(C)**, but was not statistically significant. Successful knockdown of MUC18 was confirmed via Western Blot and densitometry **(D)** in cells after 24 hours of MUC18 or control siRNA transfection. Data are presented as means ± SEMs. A single star (*) indicates p<0.05 and two stars (**) indicates p<0.001.

We first confirmed successful knockdown of MUC18 at baseline via Western Blot (Fig 5D). We performed a time course study of HRV-1B infection in human tracheobronchial epithelial cells and determined that viral load peaked 24 hours after infection, compared to other time-points (15 minutes to 2 hours). Our selection of the 24 time-point was consistent with previously published research [17–19]. Across cells from three donors, MUC18 KD cells had significantly lower viral load compared to control at 24 hours (Fig 5A). Expression of Mx1 also trended to be higher in MUC18 KD cells compared to controls, but this was not significant (Fig 5B). There was less induction of IL-8 in MUC18 KD cells compared to control cells (Fig 5C).

### MUC18 facilitates viral entry to human airway epithelial cells

**Fig 6:**
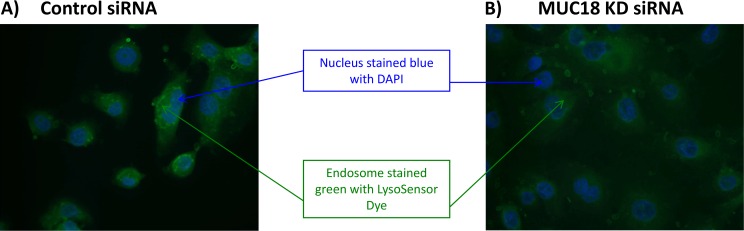
Fluorescent Green Dye to indicate endosome acidity in human tracheobronchial epithelial cells infected with rhinovirus 1B. Tracheobronchial epithelial cells from donors were seeded onto cover slips and transfected with control or MUC18 siRNA and infected with culture medium (–) or HRV-1B for 2 hours. 30 minutes prior to the end of infection, LysoSensor Dye was added to the cultures. Cover slips were removed and stained with DAPI to indicate the nucleus and mounted onto microscope slides. Control siRNA cells were more green, indicating a lower pH **(A)** and cells transfected with MUC18 siRNA were less green, indicating a higher pH **(B)**.

A fluourescent dye was used to stain the endosome based on increasing acidity. Strong green color indicates a low pH endosome, an indicator of greater acidity and viral entry. MUC18 KD cells had less green (indicating less viral entry) compared to control siRNA cells that were darker green at 2 hours (indicating a lower, more acidic pH) (Fig 6).

## Discussion

We have found that MUC18 promotes viral infections both in vivo and in vitro. In an in vivo setting, antiviral genes are significantly higher in Muc18 deficient mice, coupled with lower viral load in both BAL fluid and lung tissue. Moreover, in an in vitro setting using an air-liquid interface culture of tracheal epithelial cells isolated from Muc18 KO and WT mice, our in vivo findings were confirmed. Epithelial expression of the antiviral genes IP-10 and IRF-3 are also higher in Muc18 KO mice, even in the absence of infection, suggesting an inhibitory effect of Muc18 on antiviral gene expression.

Although MUC18 up-regulation was described in asthmatic airway epithelial cells [4], its function in lung defense against viral infection has not been investigated. Rhinovirus infection is a major contributor to asthma exacerbations, which is characterized by excessive airway inflammation, especially neutrophilic inflammation [20]. Here, by using the Muc18 KO mouse model, we clearly demonstrated that Muc18 promotes in vivo neutrophilic inflammation during rhinovirus infection because Muc18 KO mice have decreased levels of neutrophils compared to WT mice, both in percentage and absolute number. Furthermore, we found less KC production in rhinovirus-infected Muc18 KO (vs. WT) tracheal epithelial cells. How Muc18 promotes neutrophilic inflammation remains unclear. In our earlier report using a mouse model of bacterial infection [5], MUC18 enhances pro-inflammatory responses in part through NF-κB activation. Additional mechanisms warrant further investigation.

One important finding in the current study is that MUC18 inhibits lung defense against rhinovirus infection. Without MUC18, lung HRV infections are less severe as demonstrated in the mouse model. We also confirmed the in vivo finding by using a cell culture model of Muc18 KO tracheal epithelial cells, and also by using the MUC18 knockdown approach in human tracheobronchial epithelial cells. Collectively, our data suggest that MUC18 may inhibit antiviral gene (e.g., Mx1 and IP-10) expression, leading to increased load of viruses. This finding along with our discovery of MUC18’s pro-inflammatory function indicates a detrimental role of up-regulated MUC18 in diseased lungs. In individuals with up-regulated MUC18 in the airways, rhinovirus could lead to excessive neutrophilic inflammation, while impairing host defense against viruses. This may result in exacerbations of diseases such as asthma.

It is still a mystery why MUC18 promotes viral infection. One possibility is its ability to inhibit antiviral gene expression such as expression of Mx1, which represents induction of type 1 interferons [21]. We show less Mx1 expression in Muc18 KO lungs and tracheal epithelial cells, as well as in MUC18 knockdown human airway epithelial cells. We acknowledge that measuring Mx1 is not a complete approach to evaluate antiviral gene expression. Other antiviral genes such as OAS1 could also be measured to better reflect the broad spectrum of antiviral response. As interferon regulatory factors (IRFs) such as IRF-3 are critical to antiviral gene expression [22], we compared IRF-3 mRNA expression between Muc18 KO and WT tracheal epithelial cells. Interestingly, even in the absence of viral infection, IRF-3 levels are higher in Muc18 KO cells, suggesting an inhibitory effect of MUC18 on antiviral gene expression at the upstream signaling level.

Another possibility we tested is whether MUC18 promotes viral entry into the cells. We used a fluorescent dye, LysoSensor, to stain the endosome based on acidity. In MUC18 knockdown human airway epithelial cells, there is less viral entry in the early phase (e.g., 2 hours) of viral infection. Our experiment regarding the impact of MUC18 on HRV entry into human airway epithelial cells is considered exploratory. We realize that more in-depth experiments are needed to more precisely track the viral entry process by using other approaches, such as transmission electron microscopy. Although further mechanistic studies are needed to deepen our understanding of MUC18’s inhibitory function on antiviral gene expression, our current study suggests that multiple mechanisms are likely involved.

There are several potential limitations to our current study. First, we only evaluated the mice at 24 hours post infection. Although this time point is the most utilized in various publications, a time course study may be needed to better define the dynamics of pro-inflammatory and antiviral responses during rhinovirus infection. For example, although neutrophil level is significantly lower in Muc18 KO mice than WT mice at 24 hours post infection, neutrophil chemokine KC is not significantly different between the KO and WT mice. This may be explained by the fact that KC induction by an infection usually precedes neutrophil recruitment [23]. It will be interesting to measure neutrophil chemokine (e.g., KC) levels at an early time point (e.g., 4 hours) of viral infection. Second, more mechanistic studies will be planned to define how MUC18 affects the transcriptional and translational regulation of antiviral genes such as IRF-3 and Mx1.

Antiviral gene expression and inflammatory response data in Muc18 KO tracheal epithelial cell culture model appear to be more robust than those in human airway epithelial cells with siRNA-mediated MUC18 knockdown. One of the explanations is that full knockout of MUC18 in mice was a more effective model than a knockdown of the gene in humans. An interesting finding is that viral load was significantly lower in BAL fluid of Muc18 KO mice compared to wild-type mice, but such difference was not significant in the lung tissue. We believe that BAL fluid is more sensitive than lung tissue because BAL fluid collects the lining fluid of airway epithelium, which is the major site of viral infection. In the whole lung tissue, there are many types of cells that could dilute the viral signal.

In conclusion, MUC18 serves as a novel mechanism to regulate host responses to viral (e.g., rhinovirus) infection. Excessive MUC18 expression in diseased airways may contribute to virus-mediated exacerbations of lung diseases such as asthma and COPD. Targeting MUC18 likely attenuates airway inflammation and viral infection.
